# Supplementary material for: Concurrent Validity and Relative Reliability of the RunScribe™ System for the Assessment of Spatiotemporal Gait Parameters During Walking
Source: Sensors (Basel). 2024 Dec 7;24(23):7825. doi: 10.3390/s24237825 (PMC11644950; doi:10.3390/s24237825)
Supplement: Supplementary file 1 [file sensors-24-07825-s001.zip › sensors-3301663-supplementary.pdf]

Table S1 compares the ICC values obtained using the ICC(2,k) and ICC(3,k) models for the variables analyzed in the study. This comparison highlights the methodological differences between the two approaches, highlighting how the ICC(2,k) provides a more rigorous and realistic assessment of measurement consistency which represents a relevant methodological contribution compared to the more limited use of the ICC(3,k).

Table S1. Comparative analysis of gait ICC variables during walking from the OptoGait™ and RunScribe™ systems.

Measures	ICC (3, k)	ICC (2, k)
**SF (steps/min)**	0.965 ± [0.96-0.97]	0.965 ± [0.96-0.97]
**ST (s)**	0.958 ± [0.95-0.97]	0.957 ± [0.95-0.96]
**SL (m)**	0.880 ± [0.86-0.90]	0.868 ± [0.82-0.90]
**StL (m)**	0.880 ± [0.86-0.90]	0.868 ± [0.82-0.90]
**StL% (%)**	0.875 ± [0.85-0.90]	0.860 ± [0.80-0.90]
**StT (s)**	0.958 ± [0.95-0.97]	0.957 ± [0.95-0.96]
**CT (s)**	0.900 ± [0.88-0.92]	0.641 ± [-0.18-0.88]
**SwT (s)**	0.822 ± [0.79-0.85]	0.340 ± [-0.11-0.70]
